# Myxoid liposarcoma: treatment outcomes, metastatic pattern and volumetric analysis

**DOI:** 10.1007/s00066-025-02366-3

**Published:** 2025-02-03

**Authors:** Vlatko Potkrajcic, Merle Zschiegner, Maximilian Niyazi, Verena Warm, Johannes Tobias Thiel, Sandra Frantz, Christoph K. W. Deinzer, Franziska Szelat, Elgin Hoffmann, Frank Paulsen, Franziska Eckert

**Affiliations:** 1 Department of Radiation Oncology, University Hospital Tübingen, Eberhard-Karls-University of Tuebingen, Hoppe-Seyler-Str. 3, 72076 Tuebingen, Germany; 2 Department of General and Molecular Pathology and Pathologic Anatomy, University Hospital Tübingen, Liebermeisterstraße 8, 72076 Tuebingen, Germany; 3 Department of Hand, Plastic, Reconstructive and Burn Surgery, BG Unfallklinik, Schnarrenbergstraße 95, 72076 Tuebingen, Germany; 4 Department of Orthopaedic Surgery, University Hospital Tübingen, Hoppe-Seyler-Str. 3, 72076 Tuebingen, Germany; 5 Department of Medical Oncology and Pneumology, Medical University Hospital Tuebingen, Otfried-Mueller-Straße 10, 72076 Tuebingen, Germany; 6 Department of Radiation Oncology, Comprehensive Cancer Center, Medical University Vienna, Waehringer Guertel 18–20, 1090 Vienna, Austria

**Keywords:** Soft tissue sarcoma, Myxoid liposarcoma, Hyperthermia, Metastatic pattern

## Abstract

**Background:**

Myxoid liposarcoma (MLPS) is a rare subtype of soft tissue sarcoma. This entity has a specific clinical behavior, characterized with a distinct pattern of hematogenous spread, as well as with a unique radiosensitivity and chemosensitivity. Oncologic results, metastatic patterns and treatment response after multimodal therapy were evaluated in a unicentric patient cohort.

**Methods:**

Patients with myxoid liposarcoma were retrospectively analyzed in a single institution analysis (*n* = 31). Oncologic outcomes were evaluated in 28 patients with localized MLPS treated with multimodal therapy in curative intent. Metastatic pattern was analyzed in additional 3 patients with initially metastatic disease. In patients treated with concomitant MR-guided hyperthermia in the preoperative setting (*n* = 7), tumor size response was evaluated longitudinally during radio(-chemo)therapy in thermometry MRIs and before surgery (based on preoperative imaging).

**Results:**

The median follow-up was 4.1 ± 1.0 years. The most common anatomic localization was the lower extremity (78.6%). The 5‑year rates for oncologic outcomes in 28 patients treated in curative intent were 91.7% (± 8.0%) for overall survival (OS), 77.4% (± 11.0%) for local control (LC), 60.1% (± 10.6%) for distant metastasis-free survival (DMFS) and 55.4% (± 11.1%) for disease free survival (DFS). Excellent 5‑year LC (94.7 ± 5.1%) was demonstrated for the cohort excluding 5 patients treated for local recurrences. Most patients had good pathologic response (< 10% vital tumor tissue) following neoadjuvant treatment (82.4%, 14/17). However, this did not correlate with oncologic outcomes. A specific pattern of distant metastases has been observed, with predilection for soft tissues as the most common metastatic site. Furthermore, no isolated pulmonary metastases were observed. The MR analysis demonstrated a significant tumor size reduction (≥ 25%) of the initial tumor volume in 85.7% (*n* = 6/7) patients. No local recurrences and no distant metastases were observed in patients with significant MR size reduction.

**Conclusion:**

Sequential MRIs during preoperative radiotherapy of myxoid liposarcoma show distinct patterns of the known size reduction of this specific subentity. Our analysis of metastatic patterns demonstrate mostly soft tissue metastases, no patient experienced isolated pulmonary metastases.

## Introduction

Soft tissue sarcomas (STS) are a rare and heterogeneous group of malignancies accounting for about 1% of all adult malignancies [41]. Liposarcoma represent the second most common STS subtype in adult age [22], accounting for approximately 15–20% of all STS [9]. This malignancy can be defined by adipocyte differentiation [24] and is classified into five histological subtypes: well-differentiated liposarcoma, dedifferentiated liposarcoma, myxoid liposarcoma (MLPS), pleomorphic liposarcoma, and myxoid pleomorphic liposarcoma [1, 18, 24]. Despite its shared adipocytic features, these subtypes differ in their clinical behavior, treatment concepts and treatment sensitivity, driven by underlying specific biological characteristics. Therefore, subtype-tailored management of these malignancies has been of increased importance [18, 24].

MLPS account for about 30% of all liposarcomas [24]. Histologically, they are characterized by round to oval mesenchymal cells and a number of small ring lipoblasts located within myxoid stroma [24]. These malignancies usually show a unique chromosomal rearrangement and in > 95% of cases a typical translocation t(12;16)(q13;p11) is present [27], resulting in FUS and CHOP (also called DDIT 3) gene fusion [27, 31, 36]. In a smaller proportion of patients, t(12;22)(q13;q12) translocation is presented, resulting in EWSR1-CHOP (DDIT3) gene fusion [24, 27].

This malignancy represents not only histologically (and molecularly), but also clinically a distinguished liposarcoma-subtype. Unlike dedifferentiated liposarcoma, this entity usually affects younger patients with a peak incidence in the fourth and fifth decade [27]. Predominant localization are extremities [5, 22, 23]. Furthermore, these tumors are characterized by a tendency to metastasize to non-pulmonary and especially to soft tissue sites [10, 15, 40, 45]. Treatment principles are similar to those in dedifferentiated liposarcoma. Wide surgical resection represents the cornerstone of the multimodal treatment and can be complemented with additional therapy modalities in high-risk situation such as radiation therapy and chemotherapy [27, 46]. Radiosensitivity [6, 17, 32] and chemosensitivity [12, 30] are a distinguished characteristic of MLPS, often resulting in a significant volume reduction after treatment of macroscopic tumors [14].

The goal of this study was to evaluate risk factors and oncologic outcomes for patients with MLPS undergoing multimodal treatment in curative intent. Furthermore, an analysis of volumetric data was performed in patients treated with MR-guided hyperthermia with macroscopic measurable tumor (delivered during radiotherapy). Additionally, metastatic pattern and risk factors were analyzed in all patients treated in our institution (including patients with metastatic disease on initial presentation).

## Materials and methods

Clinical records of patients with MLPS were retrospectively analyzed in a single institution analysis. The study protocol was approved by the local Ethics Committee (Nr. 909/2019BO2). All adult patients with histologically confirmed MLPS treated at our institution between 2000 and 2019 were considered for analysis. The diagnosis was confirmed using either molecular-pathological analyses (FUS-CHOP/DDIT3 or EWSR1-CHOP/DDIT3 gene fusion) or reference pathology. All unclear pathological reports were reviewed by a pathologist and re-evaluated (including additional molecular analysis in unclear cases). Initial staging was carried out with contrast enhanced MRI or CT of the tumor-region and at least CT of the lungs.

Patients with high-risk localized MLPS scheduled for multimodal therapy in curative intent were included in this analysis. High risk situation was characterized by tumor size > 5 cm, subfascial localization and intermediate or high grading according to FNCLCC. Classical grading was used instead of round cell compartment, as this information was available also in cases treated several years ago. Multimodal treatment consisted of surgical resection and either preoperative or postoperative radiotherapy (3D conformal radiotherapy-3DCRT or intensity-modulated radiotherapy-IMRT). The standard dose for preoperative radiotherapy was 45.0–50.4 Gy in 25–28 fractions. For postoperative therapy a dose of 50.0–50.4 Gy was complemented with an additional boost to the tumor bed with 10–20 Gy in 5–10 fractions. Due to advanced age, one patient was treated with hypofractionated neoadjuvant radiotherapy with 25 Gy in 5 fractions [34]. Radiotherapy planning was based on a planning CT using individual positioning. Target volume contouring was performed using Monaco planning system (Version 5.11.03) or Oncentra Masterplan treatment planning system 4.3 (both Elekta AB, Stockholm, Sweden), by the aid of the diagnostic imaging. Radiotherapy treatment planning was performed by the inhouse product Hyperion 2.4.5 or the above-mentioned Monaco and Oncentra Masterplan. Radiotherapy was delivered by Elekta linear accelerators using 6 MV photons. Portal imaging and cone-beam CT were used for positioning control. Concomitant and/or sequential chemotherapy was applied in selected patients (younger patients with high-grade myxoid liposarcoma). Concomitant chemotherapy consisted of two cycles of ifosfamide (3000 mg/m^2^ on day 1 and 2, as well as on day 21 and 22 of the irradiation). Sequential chemotherapy was applied following the IAWS-protocol [19], using the combination of ifosfamide (3000 mg/m^2^ on day 1–3) and doxorubicin (60 mg/m^2^ on day 1) every 22 days for up to 3–6 cycles. Locoregional hyperthermia was delivered twice a week for 60 min over the radiotherapy course in selected patients right before or after radiotherapy fraction, using MR-guided hyperthermia, regional deep or superficial hyperthermia (BSD 2000/3 D MRI, Pyrexar Medical, formerly BSD medical corporation, Salt Lake City, UT). In combined schedules, ifosfamide was applied simultaneously with locoregional hyperthermia. Treatment decisions were made individually in multidisciplinary team meeting. Additional chemotherapy was indicated in young patients with more high-risk features and also omitted for older patients or patients in limited clinical conditions. Hyperthermia was considered in all patients with high-risk tumors. Patients with contraindications for hyperthermia were excluded (e.g. metal implants, cardiovascular disease, thrombosis).

Oncologic outcomes and risk factors (patient and tumor characteristics) were analyzed for patients with localized MLPS treated with multimodal therapy in curative intent. For the analysis of metastatic pattern, clinical data of all patients were used. Soft tissue metastases were defined as metastases in retroperitoneum, abdominal wall, abdominal cavity and muscles or subcutaneous tissue.

The analysis of tumor volumetric data was performed on consecutive MRIs acquired for thermometry during every hyperthermia treatment until the end of radiation therapy (in patients treated with neoadjuvant therapy with concomitant MR-guided hyperthermia) as well as preoperative MRI following neoadjuvant therapy. Tumor volume contouring was performed using Monaco planning system (Version 5.11.03) in all available MRIs and was expressed in cubic centimeters (cm^3^). The volume response to neoadjuvant treatment was expressed as the percent volume change comparing the last available preoperative MRI with the initial volume. Additionally, tumor size reduction was assessed using the largest measured diameter in postoperative histopathologic reports (in centimeters), compared to the largest measured diameter in pretherapeutic imaging, and was expressed as percentual change. One patient treated with 25 Gy in 5 fractions was excluded from this analysis. Tumor volume (in MR-analysis) or size reduction (in histopathologic report) of ≥ 25% was defined as a significant reduction. In case of neoadjuvant therapy, histopathological response was evaluated using postoperative pathologic reports. Good pathologic response was defined as < 10% vital tumor tissue [41].

Statistical analysis was performed with IBM SPSS Version 26. Survival times were estimated with the Kaplan Meier method and compared using the log-rank test. Means were compared by two-sided Student’s t‑test. Correlations between categorized variables were estimated using Chi-square test and Fisher’s exact test. *P* value of less than 0.05 was defined as statistically significant. *P* value less than to 0.1 was defined as a trend to statistical significance.

## Results

### Patient, tumor and treatment characteristics

A total of 31 patients with MLPS treated between 2000 and 2019 were identified. Out of them, 3 patients received palliative radiotherapy, 28 patients received multimodal therapy in curative intent (including 5 patients with localized recurrent disease previously treated with surgery only). Patient, tumor and treatment characteristics are presented in Table 1 and 2. Lower extremity was the most common tumor localization. Most patients showed at least two high-risk STS features (tumor size > 5 cm, subfascial localization, intermediate or high grading according to FNCLCC). All patients had at least one high-risk tumor feature. Negative surgical margins (R0) were achieved in most patients (*n* = 22, 78.6%), microscopically positive surgical margins (R1) were noted in 3 patients (10.7%), surgical margin status was not known in 3 patients (10.7%). In patients with localized MLPS (*n* = 28), all patients treated with concomitant chemotherapy received 2 cycles of ifosfamide. Sequential chemotherapy was delivered in median of 4 cycles (range 3–5) in 7 patients, mostly young patients with high grade tumor. Concomitant deep or locoregional hyperthermia was applied with median number of 10 treatments (range 9–12) in 13 cases. Radiotherapy feasibility was good, with all patients finishing the intended treatment.

**Table 1:** Patient, tumor and treatment characteristics

	Whole cohort (*n* = 31)	Patients with localized MLPS (*n* = 28)
**Age (years), mean**	48.5 (22–80 years)	49.5 (22–80 years)
**Sex**		
Male	22 (71.0%)	21 (75.0%)
Female	9 (29.0%)	7 (25.0%)
**Tumor size (cm)**		
⩽5 cm (T1)	2 (6.5%)	2 (7.1%)
> 5–⩽ 10 cm (T2)	10 (32.2%)	10 (35.7%)
> 10–⩽ 15 cm (T3)	8 (25.8%)	8 (28.6%)
> 15 cm (T4)	9 (29.0%)	7 (25.0%)
Missing data	2 (6.5%)	1 (3.6%)
Mean tumor size (cm)	13.2 (5.0–28.0 cm)	13.0 (5.0–28.0 cm)
**Tumor depth to superficial fascia**		
Superficial (Ta)	3 (9.7%)	3 (10.7%)
Deep (Tb)	28 (90.3%)	25 (89.3%)
**Tumor malignancy grade**		
Grade I	14 (45.2%)	13 (46.4%)
Grade II	13 (41.9%)	11 (39.3%)
Grade III	3 (9.7%)	3 (10.7%)
Missing data	1 (3.2%)	1 (3.6%)
**Number of high-risk features**		
3	14 (45.2%)	12 (42.9%)
2	13 (41.9%)	12 (42.9%)
1	3 (9.7%)	3 (10.7%)
Missing data	1 (3.2%)	1 (3.6%)
**Localization**		
Lower extremity (including the groin area)	23 (74.2%)	22 (78.6%)
Upper extremity (including the axillar area)	2 (6.5%)	2 (7.1%)
Trunk (abdominal, pelvic and thoracic area)	5 (16.1%)	3 (10.7%)
Retroperitoneum*	1 (3.2%)	1 (3.6%)

*1 tumor localized in retroperitoneum refers to recurrent tumor after initial resection of the primary tumor at another localization

**Table 2:** Therapy characteristics for patients with localized MLPS (*n* = 28)

Neoadjuvant radiotherapy*	18 (64.3%)
Adjuvant radiotherapy	10 (35.7%)
Concomitant ifosfamide	9 (32.1%)
No concomitant ifosfamide	19 (67.9%)
Concomitant hyperthermia	13 (46.5%)
No concomitant hyperthermia	15 (53.5%)
Sequential chemotherapy	7 (25.0%)
No sequential chemotherapy	21 (75.0%)

*1 patient with hypofractionated radiotherapy (25 Gy in 5 fractions) due to advanced age and ECOG

## Oncologic outcomes

**Fig. 1:**
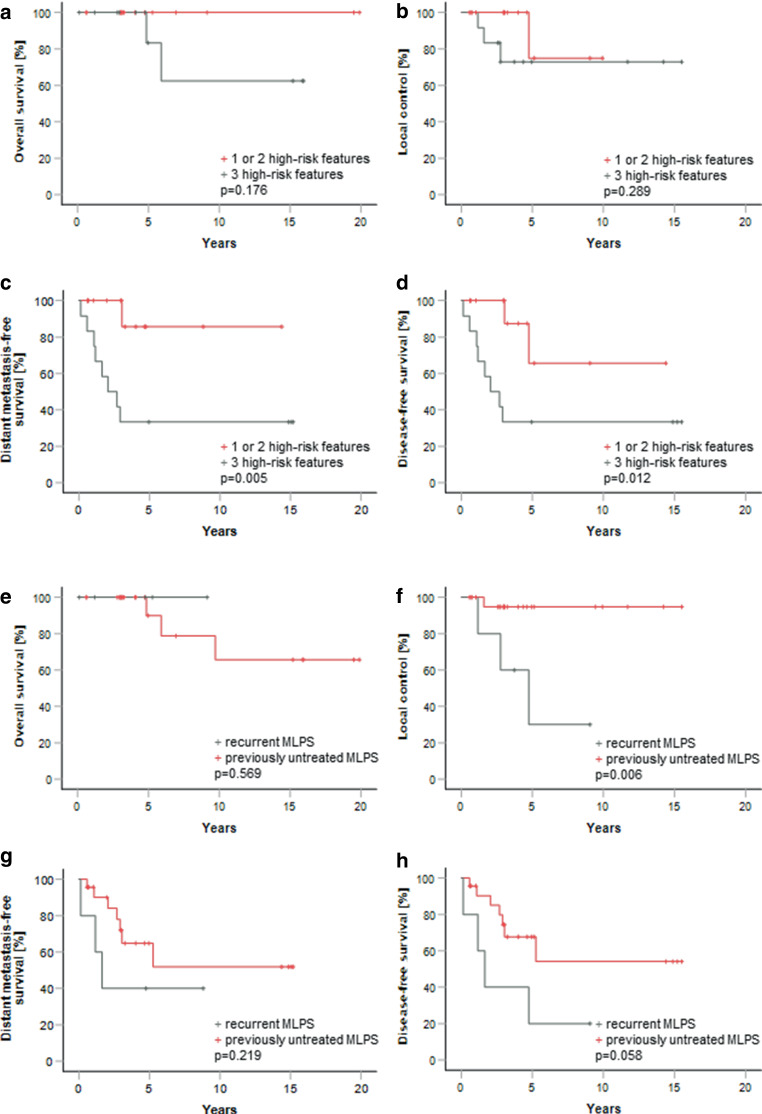
**a**, **b**, **c**, **d** Kaplan-Maier survival curves stratified by the number of tumor high-risk features. Worse DMFS and DFS were demonstrated in patients with all 3 high-risk features (*p* = 0.005 and *p* = 0.012, respectively). No difference in OS and LC was found. **e**, **f**, **g**, **h** Kaplan-Meier curves demonstrating oncologic outcomes for patients with localized MLPS, stratified by previously untreated vs. recurrent localized MLPS. Worse LC and a trend to statistical significance for worse DFS were demonstrated in patients with recurrent disease (*p* = 0.006 and *p* = 0.058, respectively). No difference in OS and DMFS was found

Oncologic outcomes were calculated for patients with localized MLPS. Median follow-up was 4.1 ± 1.0 years (mean was 6.3 years, range 0.13–19.9 years). The 5‑year rates were 91.7% (± 8.0%) for OS, 77.4% (± 11.0%) for LC, 60.1% (± 10.6%) for DMFS and 55.4% (± 11.1%) for DFS. Worse 5‑year LC was observed in patients treated multimodally for recurrent localized disease, compared to patients with previously untreated MLPS (30.0 ± 23.9% vs. 94.7 ± 5.1%, *p* = 0.006), reflecting on a trend to statistical significance for worse DFS (67.6 ± 11.1% vs. 20.0 ± 17.9%, *p* = 0.058). No difference in OS and DMFS was found (*p* = 0.569 and *p* = 0.219, respectively), results shown in Fig. 1e–h.

Worse DMFS and DFS were observed in patients with high-grade MLPS (grade 2/3 vs. grade 1) (*p* = 0.017 and *p* = 0.034, respectively), whereabout no impact on OS (*p* = 0.176) and LC (*p* = 0.358) was observed. Stratified by number of tumor high-risk features (tumor size > 5 cm, deep localization to superficial fascia and high-grade), worse DMFS (*p* = 0.005) and DFS (*p* = 0.012) were observed in patients with all 3 high-risk features (Fig. 1a–d). Four local recurrences in the whole patient cohort were observed (in one patient with R1 and three patients with R0 surgical margins). However, most local recurrences were observed in patients treated multimodally for recurrent localized disease (*n* = 3/4, 75%).

Good pathologic response after neoadjuvant therapy was observed in 14/17 patients (82.4%, data missing in one patient). Pathologic response did not correlate with treatment characteristics (concomitant chemotherapy or hyperthermia, sequential chemotherapy, data not shown). However, all patients treated with sequential chemotherapy had good pathologic response (*n* = 5). Pathologic response did not influence oncologic outcomes (OS, LC, DMFS and DFS, data not shown). Furthermore, no correlation between good pathologic response and tumor shrinkage (≥ 25%, either in volumetric analysis or in size analysis using postoperative histopathology) was observed (*p* = 0.495 and *p* = 0.255, respectively).

## Metastatic pattern for the whole cohort

**Fig. 2:**
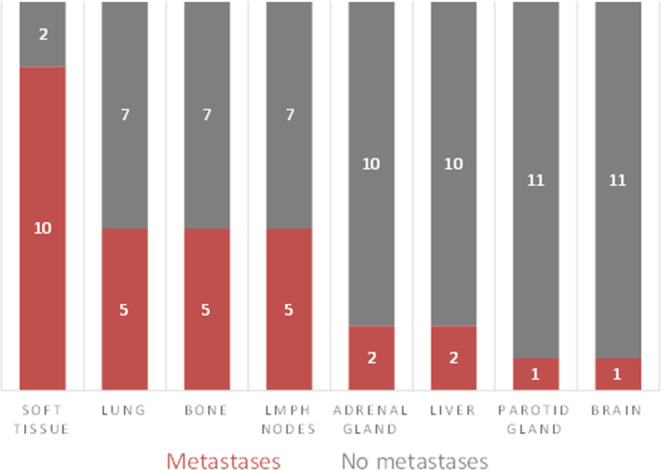
Bar graphs demonstrating the distribution of metastases in patients that developed distant metastases (*n* = 12), analyzed for the whole cohort (*n* = 31). Most common were soft tissue metastases

Distribution of metastases for the whole cohort (*n* = 31) is presented in Fig. 2. In patients with distant metastases at time of diagnosis or during follow-up (*n* = 12/31, 38.7%), soft tissue metastases were the most common site (*n* = 10/12, 83.3%), followed by lungs, bones and lymph nodes (*n* = 5/12 each, 41.6%).

In patients with soft tissue metastases, two patients had isolated soft tissue metastases. In 8/10 patients, soft tissue metastases were combined with metastases of other anatomic sites. No patients had isolated pulmonary metastases. In one patient, pulmonary metastasis was the first anatomic site of distant failure, in 4/5 patients (80%), pulmonary metastases were detected later in the disease process (after soft tissue or bone metastases). In patients with bone metastases (*n* = 5), all patients had metastases in spine and pelvis. Additional bone metastases in long bones were found in 3 patients.

## Tumor volume and tumor size during and after neoadjuvant therapy

**Fig. 3:**
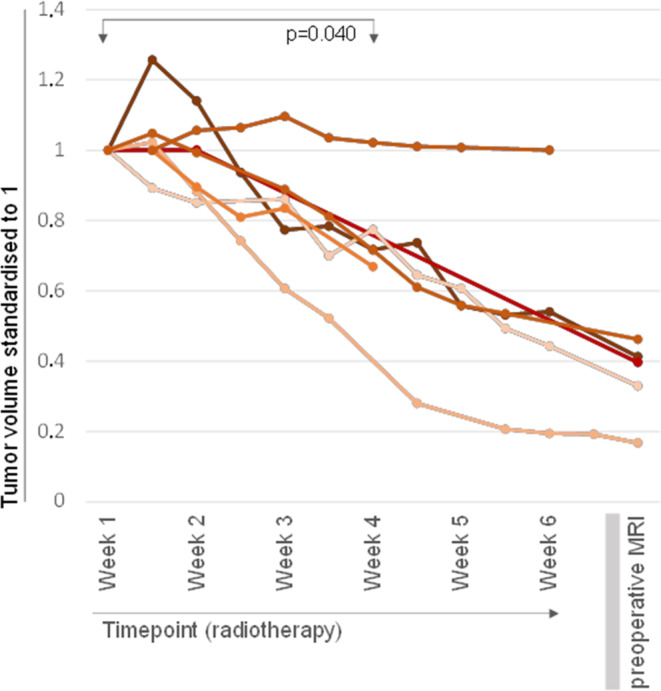
Diagram demonstrating the analysis of tumor volumetric data using all consecutive MR-imaging in 7 patients treated with neoadjuvant therapy and concomitant MR-guided hyperthermia. Relevant tumor size reduction was demonstrated in most patients (6/7 patients; 85.7%). Mean tumor volume in the first and fourth week of radiotherapy were 364.3 cm^3^ (± 228.2 cm^3^), 233.2 cm^3^ (± 187.0 cm^3^); (*p* = 0.040). In all patients with available preoperative MRI after neoadjuvant therapy, a ≥ 50% tumor downsizing was observed

**Fig. 4:**
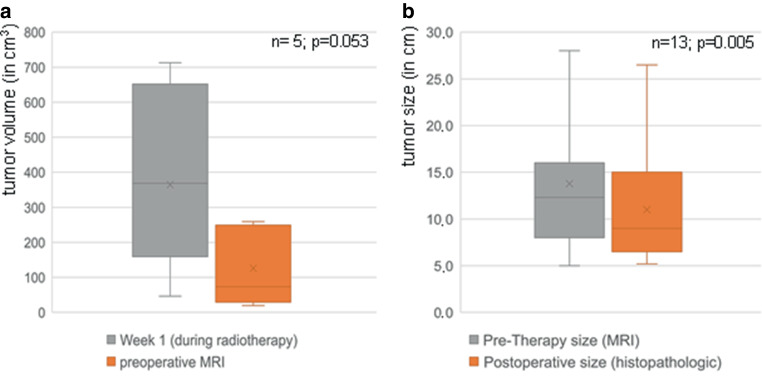
**a** Diagram demonstrating volumetric analysis in patients treated with neoadjuvant therapy with MR-guided hyperthermia. Absolute tumor volumes (in cm^3^) were compared between first week of radiotherapy and preoperative MRI (after neoadjuvant therapy). A trend to statistical significance for tumor shrinkage after neoadjuvant therapy was shown (mean tumor volume in the first radiotherapy week = 368.0 cm^3^ vs. in preoperative MRI = 126.0 cm^3^, *p* = 0.053, with limited number of patients with available data; *n* = 5). **b** Diagram demonstrating statistically significant tumor downsizing in patients treated with neoadjuvant therapy and available data (mean tumor size in pretherapeutic MRI = 13.8 cm vs. tumor size in posttherapeutic histopathologic report = 11.0 cm, *p* = 0.005; *n* = 14)

Volumetric analysis was performed in all consecutive MRIs in patients treated with concomitant MR-guided hyperthermia in addition to preoperative radiotherapy (*n* = 7) and is demonstrated in Fig. 3 and 4a. In 6/7 patients (85.7%), a significant tumor volume shrinkage (≥ 25%) was demonstrated. All patients with relevant volume shrinkage had grade 1 tumors and developed no local recurrences and no distant metastases during follow up. The only patient without tumor shrinkage had grade 2 tumor and developed local recurrence and distant metastases during the follow up. In 5/7 patients, additional preoperative MRI was available (performed after neoadjuvant therapy). In all of these patients (*n* = 5), tumor volume downsizing was ≥50%.

In patients treated with neoadjuvant therapy and with available posttherapeutic histopathologic report (*n* = 14), reduced mean tumor size following neoadjuvant treatment compared to initial diagnostic imaging was observed (13.8 ± 5.7 cm vs. 11.0 ± 5.5 cm, *p* = 0.005), Fig. 4b. A relevant tumor size reduction (≥ 25% in largest diameter) was observed in 9/14 patients (64.3%).

## Discussion

Concordant to published data [5, 6, 17], our analysis demonstrated excellent 5‑years LC-rates in patients with localized and previously untreated MLPS (94.7 ± 5.1%). Good LC-rates after combined therapy modalities may be explained with a unique radiosensitivity of these malignancies [6, 20, 32, 44]. However, in line with published data [11, 47], inferior 5‑year LC-rate was demonstrated in patients with locally recurrent disease treated multimodally in curative intent. Retrospective data report worse oncologic outcomes in STS-patients with combined risk-factors (tumor size > 5 cm, high grade, deep localization to superficial fascia) [7, 33, 38]. Thus, inferior DMFS and DFS-rates were demonstrated in patients with all 3 high-risk features as well as in patients with high grade tumors, whereas no impact on OS and LC was observed.

With 5‑years DMFS-rate of only 60.1% (± 10.6%), distant metastases represent the main pattern of therapy failure. Slightly higher DMFS-rates compared to published studies might be explained with inclusion bias, as those also included small tumors with no further risk factors, treated with surgical resection only [16, 26], whereas all patients in our study had advanced tumors with indication for multimodal treatment. Despite histological variability among STS-subgroups, a relatively uniform predilection for distant metastases has been observed in STS in general [45], with isolated pulmonary metastases as the most common site of initial recurrence in almost all subsets of STS [4, 35]. On the contrary to these findings, MLPS are characterized by an unusual and distinctive pattern of distant failure, including non-pulmonary metastases [15, 17, 32, 40, 42], with predilection for soft tissues (especially in retroperitoneum, abdominal wall and abdominal cavity) [17, 42], bones [40] and lymph nodes [45]. This unique pattern might be connected to fat-bearing areas [25], such as bone marrow, subcutaneous tissue and retroperitoneum [29]. Concordant to published data [17, 42, 45], soft tissue metastases were the most common site in our cohort. No patient had isolated lung metastases. In most patients, lung metastases were detected after soft tissue- or bone metastases, indicating that pulmonary metastases occur later in the disease process [40, 45]. Lymph nodes represent another common site and do not seem to be restricted to sentinel location, but rather represent a distant site [45]. Furthermore, one patient in our cohort had a metastasis in parotid gland, which is an uncommon metastatic site in patients with primary tumors outside the head and neck region [8]. High incidence of bone metastases has been reported in previous reports, especially with predilection for the spine [40].

Considering the distinctive and highly variable metastatic pattern, it seems therefore reasonable to perform staging and follow-up using whole body imaging [10, 17]. However, the adequate staging-method for patients with MLPS has been controversially discussed [45]. Considering the frequency of bone metastases [40] and the absence of bone destruction in bone metastases in patients with MLPS [40, 45], staging with CT scan only might be insufficient and might fail in detecting of the metastatic disease [10, 28, 45]. Therefore, especially in patients with bone pain or other symptoms indicating a suspicion for bone metastases, MRI should be considered as an investigation method of choice to detect bone metastases [28, 40]. Furthermore, considering the frequency of long bones metastases, a whole body MRI might be an adequate initial and follow up staging for this small entity [21, 43].

Previous studies indicated that MLPS are more sensitive to radiation therapy compared to other STS subtypes [6, 17, 32], with high rates of tumor shrinkage following radiotherapy [10, 14, 32]. The exact mechanism of volume reduction is not known, but might include particular cellular susceptibility, adipocyte maturation and loss of tumor stroma [6, 39]. To the best of our knowledge, this is the first study to describe the volumetric-analysis using consecutive weekly MR-imaging during radiation therapy in MLPS. Regardless heterogeneity in treatment regimens and tumor localization, excellent rates of tumor volume reduction were observed following neoadjuvant treatment, especially in patients with low grade tumors. Therefore, it seems to be recommendable to consider neoadjuvant treatment in patients where wide surgical resection is hardly manageable [3] (e.g. in very large tumors or complex anatomical locations), as a relevant volume reduction can be expected in most patients and might lead to better resectability. Furthermore, considering unique radiosensitivity and good LC-rates following neoadjuvant treatment, dose reduction of radiotherapy has been suggested. DOREMY trial demonstrated excellent oncologic outcomes as well as good feasibility after neoadjuvant treatment with 36 Gy in 2 Gy-fractions in patients with MLPS [23]. In contrast to other STS, Trabectedin seems to be specifically effective in MLPS [2]. Thus, its role in neoadjuvant setting should be evaluated in further trials.

Regardless of high rates of tumor volume/size reduction, its prognostic value seems to be unclear. No local recurrences and no distant metastases were observed in patients with significant tumor volume reduction in MR volumetric analyses. However, all of these patients had G1 tumors. One patient without response and G2 tumor was diagnosed with local recurrence and distant metastases during follow up. Due to limited number of patients, the impact of tumor shrinkage on oncologic outcomes and its prognostic value can not be assessed within this study and should be investigated in larger trials.

Concordant to published data [37], high rates of good pathologic response were observed in patients with MLPS (82.4% patients). Good pathologic response following neoadjuvant treatment was shown to be of a prognostic value in patients with STS in general [13]. A correlation between volume reduction ≥ 50% in MRI and good pathological response in patients with STS has been reported by Roberge et al. [37]. However, these observations were not confirmed in our study (with limited number of patients), suggesting a different biological behavior of MLPS compared to soft tissue sarcomas in general. Pathologic response following neoadjuvant treatment might therefore not be considered as a reliable prognostic marker for patients with MLPS. Biological tumor response to therapy might rather be reflected in changes in tumor size after neoadjuvant treatment. These questions should be investigated in further trials.

## Conclusion

Our data suggest beneficial effects of neoadjuvant radiotherapy regarding tumor size reduction in patients with MLPS. For this reason, it seems to be recommendable to apply neoadjuvant therapy in patients with localized high-risk disease, especially in cases where wide surgical resection or adjuvant radiation dose are hardly manageable. Good pathologic response following neoadjuvant therapy was demonstrated in most patients. However, its prognostic value seems to be unclear and shall be investigated in further trials, perhaps adapted to initial tumor size. Furthermore, due to an unusual and distinctive pattern of distant failure in patients with MLPS, including non-pulmonary metastases, it seems reasonable to perform adapted staging using whole body imaging (including extremities) in these patients.

## Data Availability

The datasets used and analysed during the current study are available from the corresponding author on reasonable request.

## Abbreviations

3DCRT: 3D conformal radiation therapy
CT: Computed tomography
DFS: Disease-free survival
DMFS: Distant metastasis-free survival
FNCLCC: Fédération Nationale des Centres de Lutte Contre le Cancer
Gy: Gray
IGRT: Image-guided radiation therapy
IMRT: Intensity-modulated radiation therapy
LC: Local control
MLPS: Myxoid liposarcoma
MR(I): Magnetic resonance (imaging)
OS: Overall survival
R0: Negative surgical margins
R1: Microscopically positive surgical margins
STS: Soft tissue sarcoma
